# Complete and robust magnetic field confinement by superconductors in fusion magnets

**DOI:** 10.1038/s41598-024-54165-y

**Published:** 2024-02-13

**Authors:** Natanael Bort-Soldevila, Jaume Cunill-Subiranas, Alvaro Sanchez

**Affiliations:** 1 Departament de Física, Universitat Autònoma de Barcelona, 08193 Bellaterra, Barcelona Spain; 2 Barcelona, Spain

**Keywords:** Magnetically confined plasmas, Applied physics

## Abstract

The fusion created by magnetically confined plasma is a promising clean and essentially unlimited future energy source. However, there are important problems hindering controlled fusion like the imperfect magnetic confinement and the associated plasma instabilities. We theoretically demonstrate how to create a fully confined magnetic field with the precise three-dimensional shape required by fusion theory, using a bulk superconducting toroid with a toroidal cavity. The vacuum field in the cavity consists of nested flux surfaces. The coils creating the field, embedded in the superconducting bulk, can be chosen with very simple shapes, in contrast with the cumbersome arrangements in current experiments, and can be spared from large magnetic forces between them. Because of the superconductor properties, the system will tend to maintain the optimum field distribution in response to instabilities in the plasma. We numerically demonstrate how a fully-confined magnetic field with the three-dimensional spatial distribution required in two of the most advanced stellarators, Large Helical Device and Wendelstein 7-X, can be exactly generated, using simple round coils as magnetic sources. Current high-temperature superconductors can be employed to construct the bulk superconducting toroid. This can lead to optimized robust magnetic confinement and largely simplified configurations in future fusion experiments.

One of the main challenges of our society is to produce clean energy in ways that do not damage the environment. Nuclear fusion of light nuclei—the energy of the Sun—is the most promising technology for a clean and safe solution for our long-term energy needs. Fusion requires the spatial confinement of plasma at high temperatures and pressures^1–3^. Tokamaks and stellarators are among the most advanced strategies to realize the fusion reaction; they are based on the confinement of thermonuclear plasma by toroidal magnetic fields^3–5^, because confined trajectories of magnetic fields are only possible for tori^2^.

To confine magnetic fields in toroidal geometries it is necessary to have topologically stable nested flux surfaces in the plasma volume^2^. Flux surfaces are defined as those for which the magnetic field **B** is parallel to them; field lines must lie on the toroidal surfaces, coming arbitrarily close to every point of that surface as the number of toroidal transversals goes to infinity^2,6^. The field required for plasma confinement can be realized by a rotational transform of a toroidal magnetic field so that the field has to have both toroidal and poloidal components^2,6^. The toroidal field can be created, for example, by current loops in the surface of a toroid, as in tokamaks. The required superimposed poloidal field can be produced by either a toroidal current induced in the plasma by an external ac field as in tokamaks, or by external coils as in stellarators^4,6,7^. For the latter option, two ways of twisting the magnetic field lines exist: elongating the flux surfaces and making them rotate poloidally as one moves around the toroid or making the magnetic axis non-planar^4^. Some stellarators like Large Helical Device (LHD) are based only on the first option and others, like Wendelstein 7-X (W7-X) or TJ-II, use both^4,8–12^.

The key to magnetic confinement of plasma, and therefore, to controlled fusion, is to generate the adequate magnetic flux surfaces in the volume containing the plasma and to maintain these magnetic surfaces in the event of plasma instabilities or turbulences^2,3,6,13–16^. In spite of decades of intense efforts, including clever computer-helped optimizations for coil designs^11,17,18^, in current fusion experiments a magnetic configuration that is robust enough to ensure confinement in the presence of plasma instabilities has not been achieved. This is the main reason impeding the realization of the long-sought net-energy fusion reaction^1,3,6,15^.

In this work, we demonstrate how to perfectly confine a magnetic field in a toroidal volume, with the precise shape of the desired magnetic flux surfaces. This is achieved in a toroid made of bulk superconducting material that has a cavity carved along the toroidal direction and a set of embedded coils in the poloidal one (see Figs. 1, 2). From fundamental electromagnetic theory and 3D finite-element simulations, we demonstrate that the field created by the coils is confined in the toroidal cavity and that the field distribution directly results from the shape chosen for the cavity, independently of the configuration of the coils, so that the field can be made with the exact shape of the desired flux surfaces. The system will tend to react against magnetic perturbations or instabilities by preserving the parallel-field boundary condition at the superconductor surface. The properties of superconductors and the topology make the system robustly preserve the magnetic confinement shape even when windows for accessing the plasma are incorporated into the system.

Superconductors have already been proposed to be used in fusion magnets as a set of tiles or monoliths that help shape the magnetic field created by simple coils^19^. Another recent related strategy proposes using permanent magnets instead^20^. However, in these proposals the field would be not fully confined to the desired region, and, more importantly, the field configuration is fixed, given by the spatial disposition of coils and tiles, which could not readily respond to magnetic perturbations in the plasma region. Instead, our approach considers a continuous bulk superconductor that not only fully confines the magnetic field inside the cavity volume but would react to any field modifications by inducing currents in the superconductor that will naturally preserve the boundary condition and thus the optimum magnetic shape. Our concept is reminiscent of the idea of ‘magnetic molding’ by a conducting shell^21^, which was never put in practice because of the impossibility of properly discretizing the shell into a set of conducting wires that is valid for all field shapes. In our proposal, the continuous superconducting surface responds to any field modification tending to restore the desired flux surface.

In general, magnetic fields emanate from the current loops and coils with a fixed spatial shape (dipolar at large distances). All current fusion experiments, and also particle-accelerator or magnetic-resonance magnets, have loops or coils as their magnetic sources, so they cannot avoid the stringent limitation of obtaining a field landscape resulting from the superposition of the field shape of the coils. Also, the fields of coils are extended all over the space, which prevents field confinement. In this work, we overcome these limitations by the combination of the properties of superconductors and the topology.

Here we regard superconductors as linear media with a magnetic permeability $\mu$ of near-zero value^22^ so that the magnetic induction **B** = 0 in their interior. This property is routinely applied to magnetic shielding; very good shielding of fields larger than 1 T has been achieved by bulk high-temperature superconductors (HTS) as thin as a few millimeters^23,24^. In general, the field of a magnet or a coil fully enclosed inside a superconducting shell does not leak outside the volume^25^ (with the exception of some superconductor topologies such as a toroidal one, as discussed below). In contrast, from Ampère’s law, the field of current-carrying straight wires surrounded by a superconductor cylinder always exits the enclosure. We experimentally demonstrated in^22^ this property for a straight wire surrounded by a HTS cylindrical tube.

**Figure 1:**
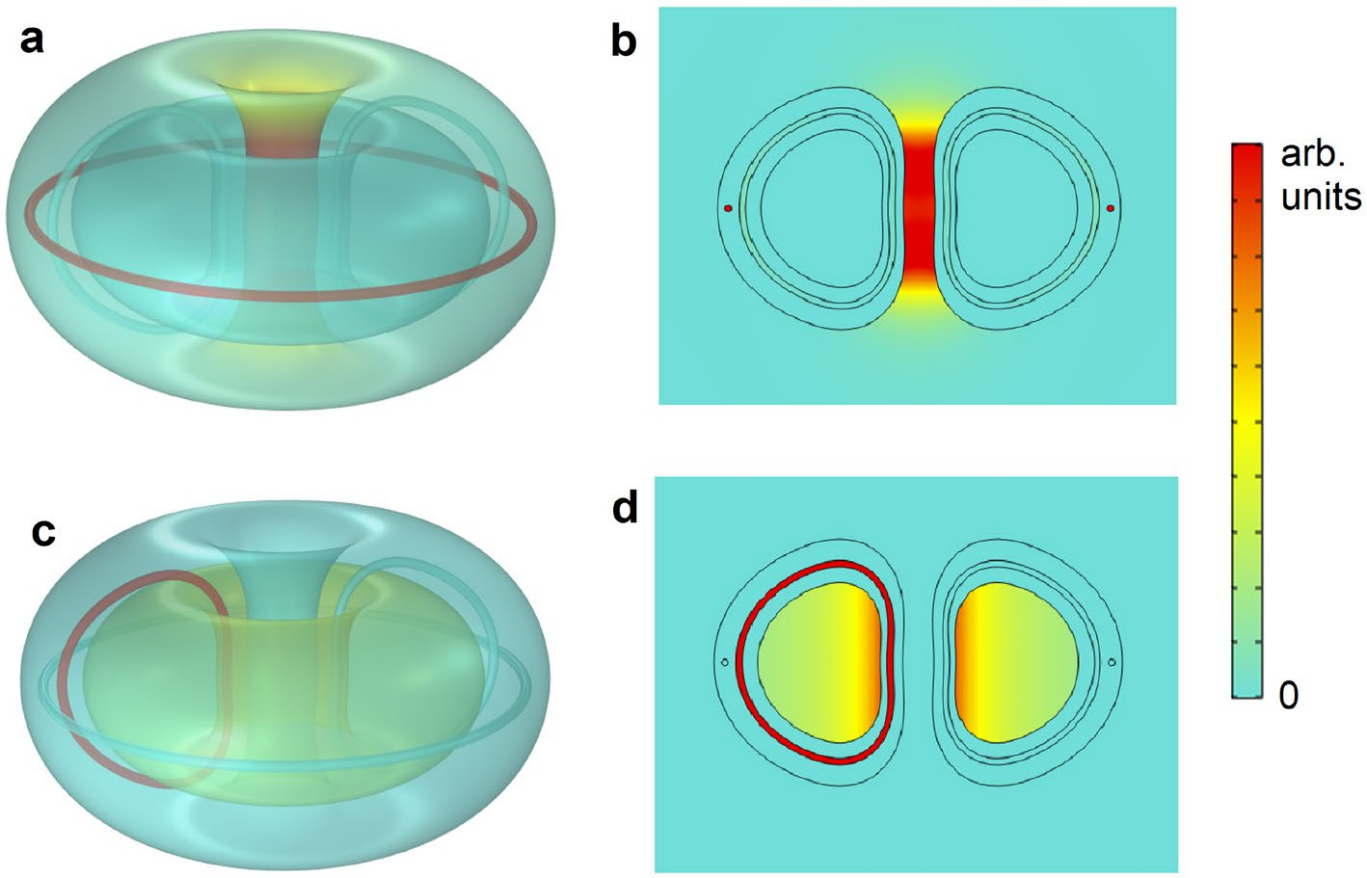
Magnetic field created by current loops embedded in a toroidal hollow superconductor. (**a**,**b**) Finite-element simulations of the 3D and 2D magnetic-field strength **B** field maps, respectively, for an embedded toroidal current loop in a bulk superconductor toroid with a cavity, for which **B** leaks outside the superconducting toroid. (**c**,**d**) 3D and 2D maps, respectively, for an embedded poloidal current loop; in this case, all the field is confined inside the toroidal cavity.

We extend these ideas to a toroidal topology. Consider a toroid made of superconducting material containing a toroidal cavity carved with the desired shape. From magnetostatics, it can be demonstrated that the field created by a current loop in the toroidal direction exits the toroid and is mostly concentrated in the central region (see finite-element simulations in Fig. 1a,b). When the loop is in the poloidal direction around the cavity, the field created is totally enclosed in the cavity, with no leak to the toroid exterior (Fig. 1c,d; see Supplementary Information for the demonstrations of all the properties).

In the case of poloidal currents (Fig. 1c,d), the magnetic boundary condition at the interface superconductor-cavity (i. e. zero perpendicular component of **B**) makes the field lines of **B** exactly follow the cavity surface, which is, by definition, a flux surface^2,6^. Since the field is parallel to the surface on the interface superconductor-cavity, and in the absence of magnetic sources, the vacuum field in the volume in the cavity consists of nested flux surfaces. The field in the plasma region is totally independent of the shape of the coils generating the magnetic field, which allows the free choice of the most convenient shape for the sources (e. g. simple round coils). The requirements for optimum magnetic confinement of plasma are naturally achieved, in contrast with actual fusion experiments in which, using very complex coils arrangements^4,6^, flux surfaces are only approximately obtained and magnetic fields are not fully confined^4,16^.

The perfect magnetic confinement is illustrated in Fig. 2. The field created by 18 current loops with the shape of the toroidal-field coils in the ITER experiment^26^ (Fig. 2a) is inhomogeneous and leaks to the exterior of the coil (Figs. 2b,c). If embedding these coils in a bulk superconductor (Fig. 2d) the field becomes fully confined in the toroid cavity, being exactly symmetric in the toroidal direction (Fig. 2e,f). The total confinement and perfect toroidal symmetry is preserved even when some coils are missing and the symmetry of the sources is lost (Fig. 2g–i), demonstrating the independence of the shapes and symmetry of the sources with respect to the actual generated field. Results in Fig. 2 highlight further robustness of our superconducting strategy; flux surfaces will keep their shape, dictated only by the toroid cavity, regardless of interruptions or fluctuations in the current feeding the coils, in contrast with current fusion experiments, where changes in the coil currents can spoil the flux surface shape^16^.

**Figure 2:**
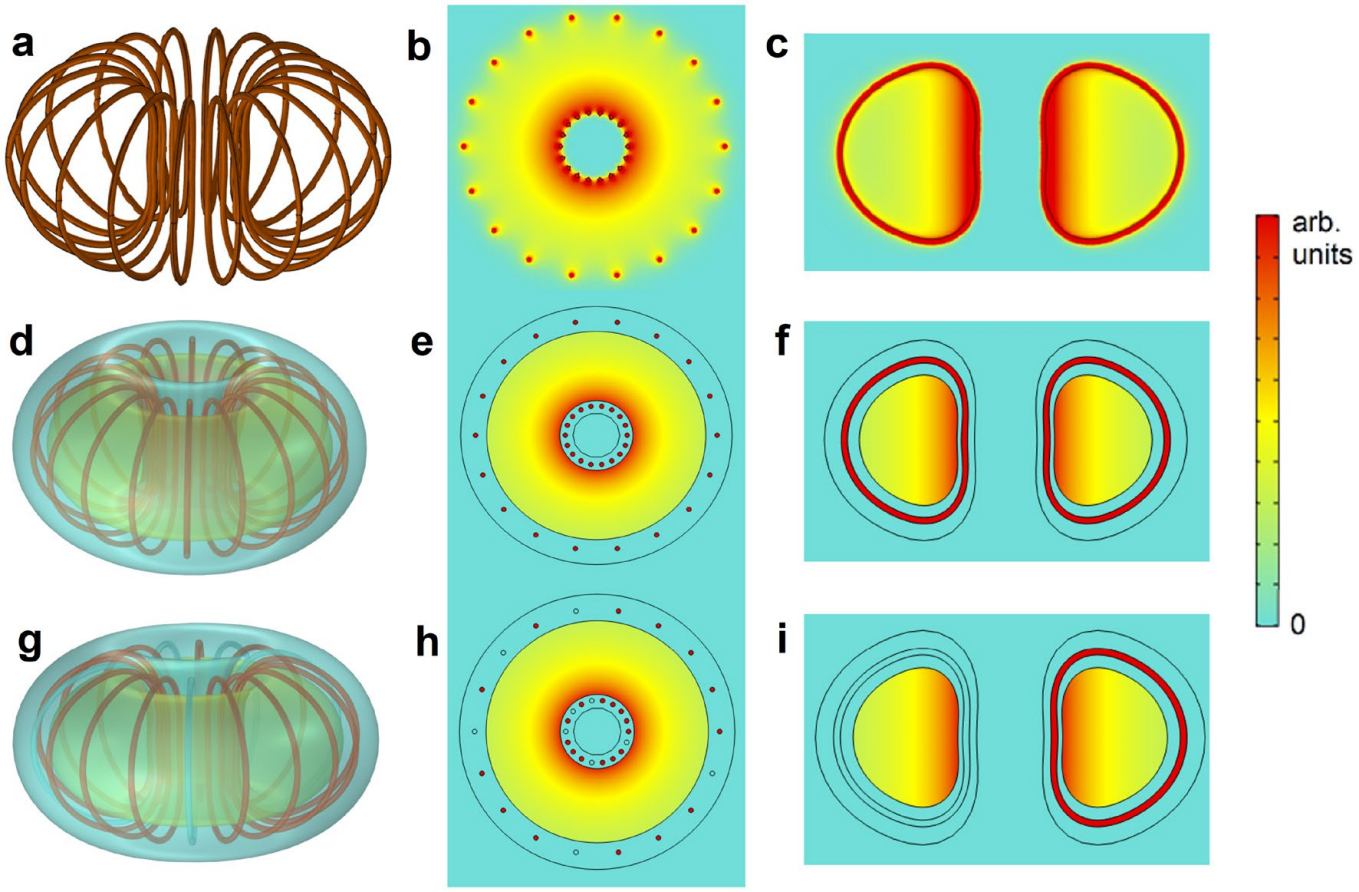
Magnetic fields in toroidal magnets. (**a**–**c**) Finite-element simulations of the magnetic-field strength distribution for 18 current loops similar to the ones used at ITER^26^. (**d**–**f**) Field distribution of the same loops when embedded inside a bulk superconducting toroid. (**g**–**i**) Field distribution when 5 out of the 18 loops are removed. The total current circulating in the previous 18 loops is now distributed in the remaining 13 loops; the obtained field is exactly the same as in (**d**–**f**).

Figure 2g–i illustrate a further relevant property: the field is zero in the poloidal voids in the superconducting bulk where there are no currents. This property was experimentally demonstrated in^22^ for parallel currents embedded in an HTS bulk cylinder with carved holes along its axis. Currents can be placed in the voids without experiencing magnetic forces from the rest of the loops; the mechanical stress is only present in the superconducting bulk, which can be made to withstand large forces, as discussed below. This may help simplify the design of fusion magnets^14,27^ and can enable the use of fragile state-of-the-art HTS tapes in the coils, with a large current capacity.

Figure 2 shows that the toroidal magnetic field created in electromagnets like the toroidal ones in ITER (Fig. 2a–c) can be made fully confined and totally homogeneous with our approach. This will be only a partial solution for the tokamak strategy, since it is left how to generate the alternating field that induces a current in the plasma to create the poloidal field. However, the perfect field confinement and the elimination of forces in the cables could be important advantages also to generate the toroidal fields in tokamaks. In the following, we concentrate on the stellarator strategy, for which the theoretical flux surfaces will be exactly obtained from the shape of the cavity in the superconducting toroid.

**Figure 3:**
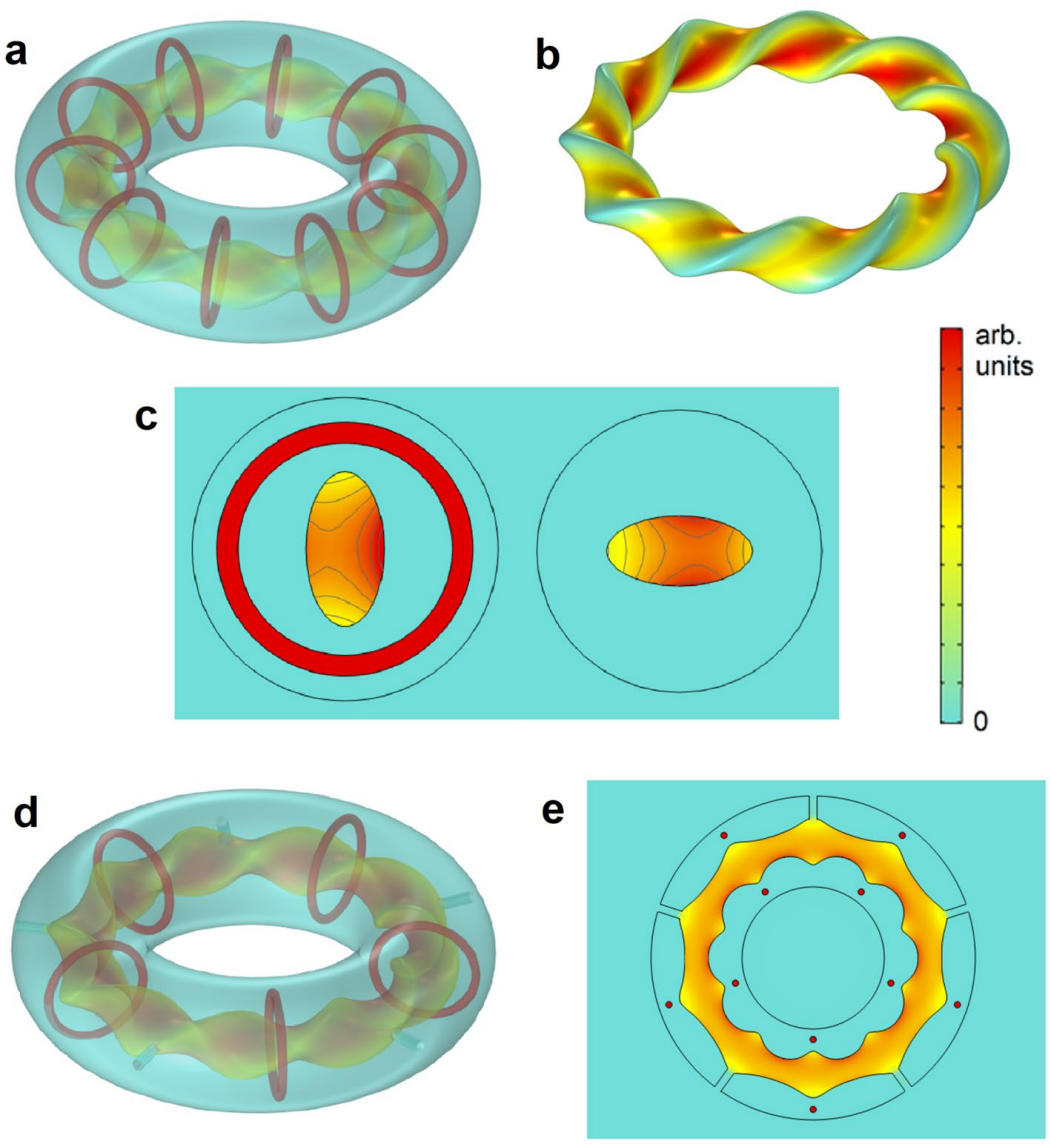
Generating magnetic flux surfaces like those in LHD experiment. (**a**) Finite-element simulations of the 3D color map of the magnetic-field strength created by a superconducting toroid with a toroidal cavity and 10 circular loops immersed in the superconductor, corresponding to the scheme of the LHD experiment. (**b**) Created magnetic flux surface at the superconducting-air boundary in the cavity. (**c**) Field profiles at two cross-sectional cuts of the cavity at different elliptical rotations. (**d**,**e**) Two views of the same configuration as in (**a**–**c**) (with 5 currents loops instead of 10), when five symmetric holes are drilled in the superconductor, in order to have ways of access to the plasma region from the exterior. The magnetic flux leakage to the holes is practically zero, thus preserving the flux shape in the cavity (see Supplementary Information for further data and discussion).

We numerically demonstrate how to achieve the ideal flux surfaces of two main current stellarator experiments. In Fig. 3a, we show that a toroidal superconductor with a carved cavity inside with the geometry of the desired flux surface and an arbitrary number of simple round coils as magnetic sources (10 in our example), reproduces exactly the ideal flux surface in stellarators like the Large Helical Device (LHD)^8,9^. The magnetic field is perfectly confined in the cavity and has the theoretically required toroidal and poloidal field components embedded within the flux surface. The profile in the cavity cross-section corresponds to the ideal one to be obtained in LHD^28,29^, with a saddle point consisting of a minimum in the direction of the minor axis and a maximum for the major axis (Fig. 3c) that rotates along the toroidal direction. Interestingly, the required field profile in the cavity is maintained even when holes are drilled in the superconductor to access the plasma region from the exterior (Fig. 3d,e). In the Supplementary Information, we demonstrate that in general, thanks to the properties of superconductors, if the holes are not big compared with the cavity size and are placed with the right symmetry, the field does not leak through them except for a small exponential decay^30^, keeping in this way the shapes of the nested flux surfaces almost intact. We numerically demonstrate in the Supplementary Information (Figs. S6–S8) how the field profile in the cavity is basically unchanged even when different numbers of holes with different dimensions are drilled in the superconductor to access the plasma region.

**Figure 4:**
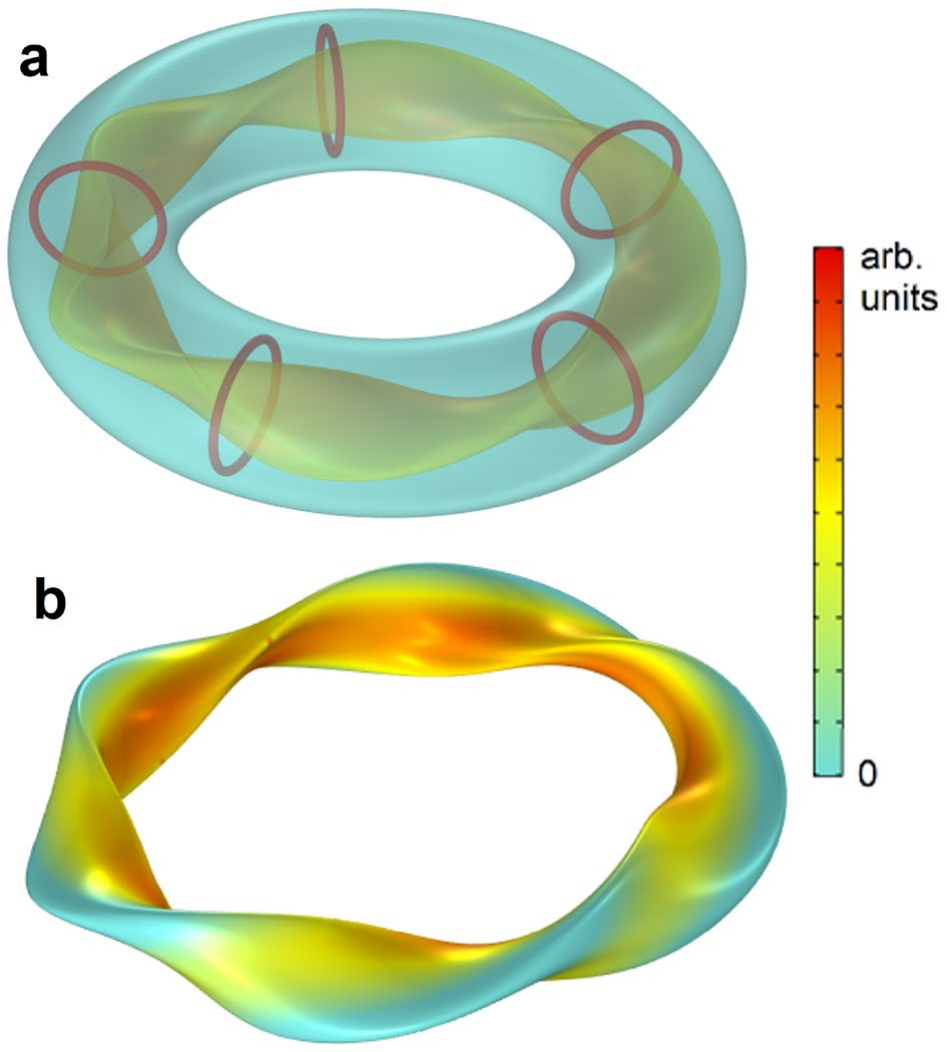
Generating magnetic flux surfaces like those in the W7-X experiment. (**a**) Finite-element simulations of the 3D color map of the magnetic-field strength created by a superconducting toroid with a toroidal cavity and 5 circular loops immersed in the superconductor, corresponding to the scheme in the W7-X experiment. (**b**) Created magnetic flux surface at the superconducting-air boundary in the cavity.

We further confirm our ideas in a geometry inspired by the recently constructed W7-X stellarator, which introduces an extra parameter that makes the magnetic axis non-planar^4^. Figure 4 shows that also in this case the obtained flux surfaces correspond to the ideal surfaces theoretically designed for stellarators like W7-X^10–12^.

With the results in Figs. 3 and 4, we demonstrate how a fully confined field with the 3D magnetic configuration required by the theory can be achieved with our approach, in the most advanced geometries currently developed in plasma fusion experiments and using simple round coils as sources. In contrast, the actual LHD and W7-X experiments have extremely cumbersome coil arrangements, designed after complex optimization processes^11,17^. They achieve only an approximation to the ideal flux surface, and the magnetic field is not confined within the flux surface but it leaks outside. Also, in our approach, the magnetic forces in the current-carrying coils are greatly reduced or even eliminated. Because the shape of the field directly results from the chosen cavity carved in the superconductor, not only the geometries corresponding to current fusion experiments but also any other geometries suggested by theory, like a recent proposal for quasisymmetric stellarators^31^, could be implemented following our ideas. These properties will make the system especially robust to magnetic fluctuations in the cavity. Magnetic instabilities will be smeared out at the outmost flux surface because any perturbation will induce superconducting currents to preserve the boundary condition of field tangential to the surface. This will act as a self-recovering mechanism. In contrast, in current experiments, magnetic disturbances interacting with the field created by fixed coils may result in runaway processes leading to loss of plasma pressure and confinement and even damage to the container walls^32^.

To implement our ideas in actual devices, one would need to address situations that are not considered in our simplified assumptions, such as imperfections in the flux surfaces, magnetic field disturbances due to currents in the plasma arising from the pressure distribution, or enabling a space to accommodate cryogenics, fuelling systems, or exhaust systems. Moreover, bulk superconducting materials should be available with the desired properties. We discuss both issues next.

We have considered only vacuum fields for the creation of nested flux surfaces. In actual settings, transport plasma processes, including turbulence or collisions, may give rise to currents like Pfirsch–Schlüter currents. These currents, induced by the pressure profile, might distort the vacuum magnetic field leading to imperfect flux surfaces, and consequently letting the plasma escape. Actually, in general, constraining the outermost flux surface does not fully guarantee closed flux surfaces within the plasma volume. Besides, fixed-boundary instabilities can occur even if the plasma boundary is fixed. The latter issues, which are general concerns for all fusion magnets proposals, imply that having nested flux surfaces is a necessary condition for fusion, but not a sufficient one, and this has to be taken into account in any future design.

Other possible practical issues are the actual location, support, refrigeration of the superconductor, and the accessibility to the interior of the sealed bulk toroid. In actual fusion magnets, there is some distance between the superconducting coils and the outer plasma volume. This empty volume is needed for two main reasons: to protect the superconductors and all the external equipment from the plasma heat, which is ultimately the goal of magnetic confinement, and to accommodate divertors, tritium breeding, lithium blankets, cryogenics, etc. If all these components have no magnetic parts, they will not distort the vacuum field in the cavity. The space between the inner superconducting surface and the plasma volume should be compatible with dealing with heat removal and with the presence of neutron wall loadings, although the details on how this would be implemented in detail are beyond the scope of this work. The solution we propose is to place the superconducting bulk with the embedded coils in the same volume that now occupies the complex set of coils in current stellarators. This arrangement generates a magnetic field within the cavity with a magnitude order similar to that of the bare current stellarator coils for the same currents. Field lines do not get compressed when the cavity has a smaller minor radius (as discussed in Supplementary Information). Instead of having a complex set of coils (more than 50 bizarrely twisted coils in Wendelstein 7-X) with their supports, we put in the same volume a much simpler set of round coils surrounded by the bulk superconductor with the tailored inner surface. Therefore, since it occupies the volume now taken by the superconducting coils, the cryogenics needed to cool the bulk superconductor could benefit from the same engineering solutions currently used. Also, this location ensures us that there would be basically the same distance (around 1 m for Wendelstein 7-X) between the coils and the boundary of the plasma region as in current devices, with the important advantage that our strategy of using a tailored-shaped bulk superconductor makes it always tending to preserve the vacuum field shape in response to magnetic fluctuations. In contrast, these fluctuations cannot be dealt with easily with the fixed set of coils employed in the present stellarators, so they can lead to loss of plasma pressure. Our general idea can be extended to include cavities with more complex shapes than the simple toroidal cavity mentioned above. For instance, we could consider subcavities connected to the main cavity through small openings. In this configuration, the magnetic field in the main cavity would remain basically intact, while the subcavities can be utilized to place the divertors. With such subcavities, one could potentially create open field lines for the outermost flux lines, allowing them to enter the subcavity and effectively remove the heat and ash produced by the fusion reaction, all while maintaining the central magnetic island in the main cavity where the reaction occurs. Since we have demonstrated that our embedded coils would experience far fewer forces than the present coils and also that some holes can be made in the bulk without affecting its properties, one can easily devise simple ways to support our coils with light structures that can pass through holes in the superconducting bulk. Additionally, the holes can provide accessibility to the sealed bulk superconducting container, allowing, for instance, the installation of systems to provide heating to the plasma, or other systems that need to come from the exterior into the container. Finally, as discussed next, the bulk superconductor can be made in pieces so that it can be easily dissembled for maintenance tasks.

The superconductors to be used in the superconducting toroid need to operate at magnetic fields of a few tesla, to withstand large forces, to be able to form large volumes, and to allow finely shaped cavities to be carved in them. Several families of state-of-the-art bulk HTS fulfill these requirements. Actually, bulk superconductors are being currently used in technologies like high-performance electrical motors, superconducting bearings, flywheel energy storage, and levitation trains^33^. Bulk superconductors of the family RE-Ba-Cu-O, where RE are rare-earth elements like yttrium or gadolinium, have shown exceptionally good superconducting properties at large fields^34^. Regarding mechanical properties, bulk HTS have been shown to withstand Lorentz forces arising from applied fields up to 7 T^35^. Even higher fields can be endured by reinforcement of the bulks by carbon fiber^36^ or stainless steel^35^. At high applied fields, some field and current will penetrate the superconductors^37^, making them depart from the ideal assumed behavior, $\mu =0$. However, the field penetration can be small for the best materials. The effective penetration depth at applied fields $B_{\textrm{a}}$ is approximately $\lambda _{\textrm{eff}} = B_{\textrm{a}}/(\mu _0 J_{\textrm{C}})$^37^. The critical current density of RE–Ba–Cu–O HTS is larger than 10^8^ A/m^2^ at fields as large as 5 T at 77 K, and it increases at lower temperatures^38^. Assuming $J_{\textrm{C}}\sim 4 \times 10^{8}$ A/m^2^, a field of 5 T would correspond to a field penetration of about 1 cm, which would not be a large departure from the ideal behavior in a few-meters fusion device. MgB_2_ and Bi–Sr–Ca–Cu–O families also present good properties at low temperatures^23^. In particular, bulk MgB_2_ has the advantage of having a lower cost compared to RE–Ba–Cu–O or Bi–Sr–Ca–Cu–O bulk superconductors. All these superconductors are ceramics or metals. They can be machined with detail into desired geometries. Such finely shaped superconductors are currently in use, for example in shielding devices^39^ and fault-current limiters^40,41^. The best properties of bulk HTS have been obtained in pellets of up to tenths of centimeters; a large structure in the form of a toroid could be constructed by assembling a number of these pellets. In^22^ we demonstrated properties analogous to those in this work using a Y–Ba–Cu–O cylindrical tube made of ten stacked disk pieces, reproducing very well the behavior theoretically predicted for a solid piece. The experimental results in^22^ indicate that the flux leakage through the contact surfaces in the superconductor assembly can be regarded as small. The fact that the superconductor is made of different pieces instead of a solid block also helps avoid flux conservation problems when cooling the device. Moreover, it has been demonstrated that radiation effects will not present a serious challenge for the use of high-temperature superconductors in fusion magnets^42,43^.

In this work, we have introduced an approach for fusion magnets that fully confines magnetic fields in a toroidal volume carved in a bulk superconducting toroid, with a set of important properties: (1) the magnetic field confined in the cavity has the shape of nested flux surfaces (disregarding imperfect optimizations or plasma instabilities); (2) no field is leaked outside the cavity, which can protect the electronic equipment in the environment of the reactor; (3) the shape of the coils energizing the magnetic fields is totally independent of the final field distributions, so that the most convenient shapes, e. g. round coils, can be employed; (4) magnetic forces between coils can be very much reduced, allowing the use of novel fragile HTS tapes; (5) the vacuum field shape is robustly preserved even when windows are drilled to access the plasma region; (6) the superconductor is always reacting to keep the boundary conditions at its surface, which may be helpful in the case of magnetic disturbances in the plasma. Beyond several applications where the shaping of toroidal magnetic fields could be interesting, our approach, combining the unique properties of superconductors with topology, can be relevant in the next generation of fusion magnets to help achieve the goal of a clean and essentially unlimited source of energy.

### Supplementary Information

Supplementary Information.

## Data Availability

All data is available in the manuscript or in the Supplementary Information.
