# Supplementary material for: Complete and robust magnetic field confinement by superconductors in fusion magnets
Source: Sci Rep. 2024 Feb 13;14:3653. doi: 10.1038/s41598-024-54165-y (PMC10864317; doi:10.1038/s41598-024-54165-y)
Supplement: Supplementary file 1 — Supplementary Information. [file 41598_2024_54165_MOESM1_ESM.pdf]

Supplementary Information: 'Complete and Robust Magnetic Field Confinement by Superconductors in Fusion Magnets'

Natanael Bort-Soldevila,[1] Jaume Cunill-Subiranas,[1] and Alvaro Sanchez[2]

[1] *Departament de Física, Universitat Autònoma de Barcelona, 08193 Bellaterra, Barcelona, Catalonia, Spain*

[2] *Independent researcher*

Contents:

1. **Methods.**
2. **Theoretical demonstration of the properties.**
3. **Drilled torus.**

1. Methods

Numerical simulations were performed using the program COMSOL Multiphysics 5.6 with the magnetic fields (mf) physics interface, which solves the equations $\nabla \times \mathbf{H} = \mathbf{J}$, $\mathbf{B} = \nabla \times \mathbf{A}$ and $\mathbf{J} = \sigma \mathbf{E}$ with boundary conditions $\mathbf{n} \times \mathbf{A} = 0$. The simulations were performed in the stationary state, using 3D spatial dimensions. The simulation mesh selected had a maximum element size of 0.08m, minimum element size of 0.002m, a maximum element growth rate of 1.3, curvature factor of 0.4 and a resolution of narrow regions of 0.7. The total calculation space was set to a 5m side length square with the relevant region of study of around 2m. For the relative permeability of the superconducting material, a value of $2 \cdot 10^{-7}$, in SI units, was typically used; when using other similar values no significant changes occurred in the results.

2. Theoretical demonstration of the properties

Magnetostatic principles

In magnetostatics, the electric and magnetic fields decouple, simplifying Maxwell equations to

$$\nabla \times \mathbf{H} = \mathbf{J}\,, \tag{1}$$

$$\nabla \cdot \mathbf{B} = 0\,, \tag{2}$$

where $\mathbf{H}$ is the magnetic field, $\mathbf{B}$ the magnetic induction, and $\mathbf{J}$ the free current density. However, in this work we refer to $\mathbf{B}$ as the magnetic field, for simplicity. Due to (2), $\mathbf{B}$ can always be expressed as the curl of the potential vector $\mathbf{A}$,

$$\mathbf{B} = \nabla \times \mathbf{A}\,. \tag{3}$$

Equation (1) can be rewritten as its integral form,

$$\oint_C \mathbf{H} \cdot \mathrm{d}\mathbf{l} = \int_S \mathbf{J} \cdot \mathrm{d}\mathbf{a}\,, \tag{4}$$

where $\mathbf{J}$ is the free current density threading a surface S generated by any closed line C,

and $\mathrm{d}\mathbf{l}$ and $\mathrm{d}\mathbf{a}$ are the length and area differentials, respectively. This implies that, in the Coulomb gauge ($\nabla \cdot \mathbf{A} = 0$), within a spatial region containing no free currents the Laplace equation,

$$\nabla^2 \mathbf{A} = 0\,, \tag{5}$$

is fulfilled (understanding that this is valid for each component of $\mathbf{A}$).

In any interface separating two media, two boundary conditions result from (1) and (2), which are, respectively,

$$\mathbf{n} \times (\mathbf{H_1} - \mathbf{H_2}) = \mathbf{K}\,, \tag{6}$$

$$\mathbf{n} \cdot (\mathbf{B_1} - \mathbf{B_2}) = 0\,, \tag{7}$$

being $\mathbf{K}$ the free surface current density and $\mathbf{n}$ a unit vector perpendicular to the interface which separates regions 1 and 2. This means that the component of $\mathbf{B}$ perpendicular to the surface is always continuous.

Interlaced cavities

Consider a bulk superconducting torus with two interlaced toroidal cavities carved inside it, one empty and the second one with a current loop within it. By interlaced we understand that one threads the other, and vice versa (like the wire and the biggest toroidal cavity in Fig. 1(c,d)). In the following, we demonstrate that the field in the empty cavity has the shape of a set of nested magnetic flux surfaces.

The magnetic field at the surface of the inner cavity will be always parallel to that surface, since (7) leads to

$$\mathbf{n} \cdot \mathbf{B_{SC}} - \mathbf{n} \cdot \mathbf{B_{cavity}} = 0 \implies \mathbf{n} \cdot \mathbf{B_{cavity}} = 0\,, \tag{8}$$

because $\mathbf{B_{SC}} = 0$. This forces the magnetic field to be contained in a flux surface with the carved shape. This particular flux surface is called the boundary flux surface. Taking into account that there are no magnetic poles or currents inside the cavity, Laplace's equation (5) is fulfilled, so, fixed the boundary conditions, a unique solution exists. Therefore, there will be magnetic flux surfaces remaining one inside the other surrounding what is called the magnetic axis. In fusion, these nested flux surfaces are needed to perfectly confine the particles inside the reactor and, neglecting the field of the plasma currents, they can be

obtained exactly by using our approach.

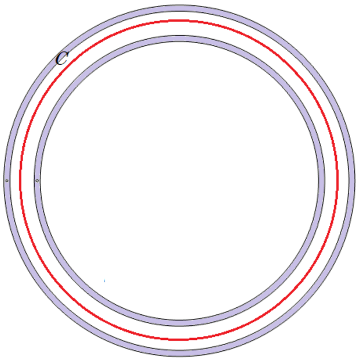

FIG. S1: Horizontal cross-section of a torus with a toroidal cavity and an interlaced embedded perpendicular current wire (left of the torus). A red curve C is drawn centered at the center of the torus to perform Ampère's calculation.

To demonstrate that the magnetic field $\mathbf{B} = \mu\mathbf{H}$ inside the cavity is not zero, Ampère's law (4) is used in a particular example of a circular toroidal cavity, with one loop of current I embedded in the superconductor (SC). A cut in the horizontal plane of this hollow torus is plotted in Fig. S1 where a red curve C is traced inside the cavity. This curve can be drawn as a circle of radius ρ centered at the center of the torus. It is always parallel to both sides of the walls of the cavity, so $\mathbf{B}$ will always be tangential to the curve C. The strength of $\mathbf{B}$ should be constant all along the line, due to the symmetry of the problem. Then,

$$\oint_C \mathbf{B} \cdot \mathrm{d}\mathbf{l} = |\mathbf{B}| \oint_C \mathrm{d}l = |\mathbf{B}| 2\pi\rho = \mu \int_S \mathbf{J} \cdot \mathrm{d}\mathbf{a} \,. \tag{9}$$

Since the surface S created by curve C has a total net current of I threading it, the magnetic field inside the cavity is not zero and has a value

$$|\mathbf{B}| = \frac{\mu I}{2\pi\rho} \,. \tag{10}$$

For any other cavity shape and any number of current loops embedded in the SC, the magnetic flux will not be zero, as long as the total net current I of all the loops has a non-zero value. Note that the magnitude of the magnetic field inside the cavity does not depend on the minor cavity radius, just on the current intensity I and the polar coordinate ρ. Shrinking uniformly along the entire toroidal cavity does not compress the magnetic field lines, as one might intuitively think. Therefore, with the same net intensity and dimensions of the toroid,

the same magnetic field magnitude will be obtained in the cavity area, independently of the volume occupied by the bulk superconductor.

Non-interlaced cavities

On the other hand, if the cavity and the embedded current wire are not interlaced (for example, like the current loop and the biggest toroidal cavity of Fig. 1(a,b)), $\mathbf{B}$ will not permeate into the cavity ($\mathbf{B_{cavity}} = 0$). Now we demonstrate that the field within the cavity is zero.

From the mathematical property

$$\oint_C \mathbf{B} \cdot \mathrm{d}\mathbf{l} = 0\,, \forall C \subset \mathcal{R} \iff \mathbf{B}_{\mathcal{R}} = 0\,, \tag{11}$$

where $\mathcal{R}$ is the entire closed spatial region in which $\mathbf{B}$ could be non-zero (i.e. $\mu \neq 0$, the cavity in our case), since there are no free currents threading a surface S generated by any possible closed line C inside the cavity, the magnetic field $\mathbf{B}$ will be always zero at every point of the cavity.

This zero value of the magnetic field leads to an absence of forces between current cables placed at different non-interlaced cavities, as discussed in the main manuscript.

Around the torus hole

Another interesting property takes place when interlacing the two cavities (one containing the current wire and the other empty) around the torus hole of the SC, as shown in Fig. S2. The magnetic field of the current wire can permeate into the other cavity and outside the SC torus. In this case, the magnetic flux also permeates outside the SC because the current wire is embedded around the SC torus hole.

This particular phenomenon allows us to modify the magnetic field inside the cavity and outside the torus simultaneously. Applied to toroidal or other kind of topologies, it can lead to novel ways to shape the magnetic field and new applications.

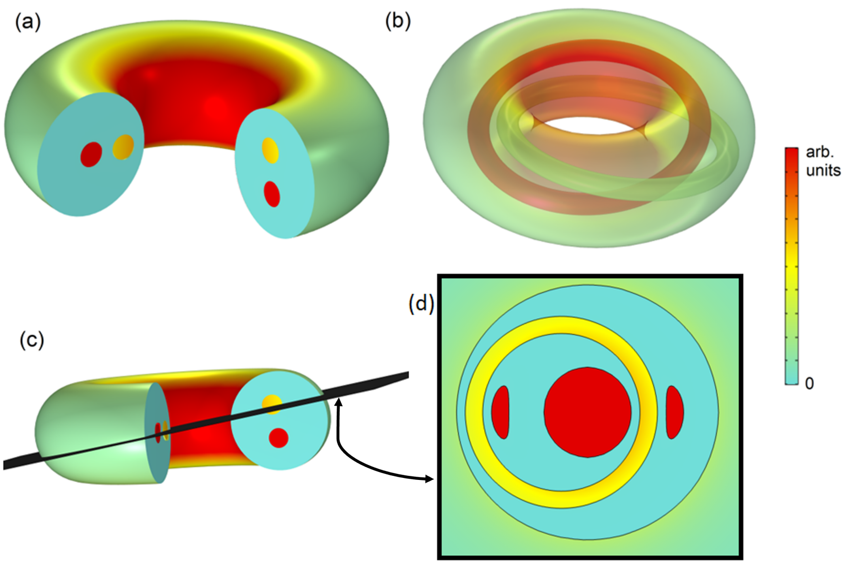

FIG. S2: (a), (b), (c) and (d), Finite-element calculations of the magnetic field strength $|\mathbf{B}|$ for a superconducting torus with two interlaced cavities, one with a current wire and another without it, both around the torus hole. The magnetic field penetrates the empty cavity and outside the SC torus. In (d), we represent the cut-plane shown in (c), displaying the entire interior of the empty cavity. This plane is parallel and intersects with this empty toroidal cavity. The red cavity corresponds to the one containing the embedded current.

3. Drilled torus

In actual fusion magnets, some holes would be needed to access the inner cavity of the torus. These holes could be drilled into the cavity by carving a tunnel connecting the outside of the SC torus and the inner cavity. Depending on where they are drilled, different outcomes of the magnetic field of the system can occur.

Field leakage

Here we demonstrate the conditions needed for no magnetic-field leakage through the holes. If the system of coils and holes is as the one in Fig. S3(a), where there is symmetry between coils, holes, and intensities around the coils, the magnetic field along the drilled holes will decay exponentially on first order, and the magnetic field inside the cavity will maintain the axisymmetric rotational symmetry. This axisymmetry is slightly locally broken at nearby distances to the hole, but without perturbing the global magnetic field. In such conditions, there is no major breaking of the magnetic confinement.

To demonstrate this effect we consider a simplified system, shown in Fig. S3(a), with two coils (with the same current) and two holes placed at symmetric positions. In this symmetric case, the magnetic field inside the cavity would be axisymmetric, since any invariant transformation of the system has the same solution for the magnetic landscape. For example, a 180º rotation around the central torus axis remains the system unchanged and thus the same solution must result. The hole dimensions are also an important factor for the uniformity of the field in the cavity. The magnetic field decays across the hole reminding us of the field decay due to a dipole in a superconductor hole (see reference [31] of the main manuscript), where the magnetic field decays exponentially at large distances. To have an exponential decay of the magnetic field across the hole in our system, it must have a longitude-diameter quotient as large as possible. Another factor determining how locally the field is distorted around the hole is the quotient between the cavity diameter and the hole diameter. The bigger this quotient is, the more local this effect will be and the less distorted the magnetic field lines will be near the hole.

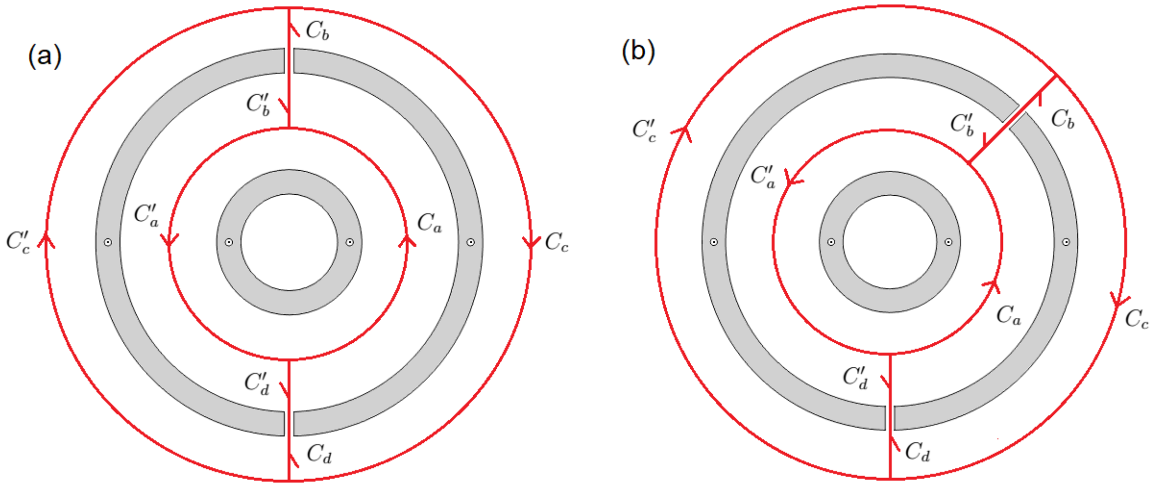

FIG. S3: (a), Horizontal cross-section of a symmetrical drilled torus. Two red closed curves C and C' are drawn to perform Ampère's demonstration. (b), Horizontal cross-section of a clearly asymmetrical drilled torus. The red curves C and C' are drawn too.

If this rotational symmetry is broken, for example, by moving one hole like in Fig. S3(b), the magnetic field inside the cavity will lose its axisymmetry, and the field will leak outside the cavity. This phenomenon can be understood using Ampère's law (4). Considering the

two closed curves

$$C = C_{\rm a} + C_{\rm b} + C_{\rm c} + C_{\rm d} \ ,$$
$$C' = C'_{\rm a} + C'_{\rm b} + C'_{\rm c} + C'_{\rm d} \ ,$$

drawn in Fig. S3, we define the line integrals for the magnetic field along these lines as

$$\begin{aligned} \mathcal{I} &= \int_{C_{\rm a}} \mathbf{B}_{\rm a} \mathrm{d}\mathbf{l} + \int_{C_{\rm b}} \mathbf{B}_{\rm b} \mathrm{d}\mathbf{l} + \int_{C_{\rm c}} \mathbf{B}_{\rm c} \mathrm{d}\mathbf{l} + \int_{C_{\rm d}} \mathbf{B}_{\rm d} \mathrm{d}\mathbf{l} = \mathcal{I}_{\rm a} + \mathcal{I}_{\rm b} + \mathcal{I}_{\rm c} + \mathcal{I}_{\rm d} \ , \\ \mathcal{I}' &= \int_{C'_{\rm a}} \mathbf{B}'_{\rm a} \mathrm{d}\mathbf{l} + \int_{C'_{\rm b}} \mathbf{B}'_{\rm b} \mathrm{d}\mathbf{l} + \int_{C'_{\rm c}} \mathbf{B}'_{\rm c} \mathrm{d}\mathbf{l} + \int_{C'_{\rm d}} \mathbf{B}'_{\rm d} \mathrm{d}\mathbf{l} = \mathcal{I}'_{\rm a} + \mathcal{I}'_{\rm b} + \mathcal{I}'_{\rm c} + \mathcal{I}'_{\rm d} \ . \end{aligned} \tag{12}$$

Therefore, by using Ampère's law (4) and considering both coils with the same intensity I we can obtain this equation system

$$\begin{cases} \mathcal{I}_{\rm a} + \mathcal{I}_{\rm b} + \mathcal{I}_{\rm c} + \mathcal{I}_{\rm d} = \mu I \\ \mathcal{I}'_{\rm a} + \mathcal{I}'_{\rm b} + \mathcal{I}'_{\rm c} + \mathcal{I}'_{\rm d} = \mu I \\ \mathcal{I}_{\rm b} + \mathcal{I}'_{\rm b} = 0 \\ \mathcal{I}_{\rm d} + \mathcal{I}'_{\rm d} = 0 \\ \mathcal{I}_{\rm a} + \mathcal{I}'_{\rm a} = 2\mu I \\ \mathcal{I}_{\rm c} + \mathcal{I}'_{\rm c} = 0 \end{cases} \Longrightarrow \begin{cases} \mathcal{I}_{\rm a} = \mu I - \mathcal{I}_{\rm b} - \mathcal{I}_{\rm c} - \mathcal{I}_{\rm d} = \mu I - K \\ \mathcal{I}'_{\rm a} = \mu I + \mathcal{I}_{\rm b} + \mathcal{I}_{\rm c} + \mathcal{I}_{\rm d} = \mu I + K \\ \mathcal{I}'_{\rm b} = -\mathcal{I}_{\rm b} \\ \mathcal{I}'_{\rm c} = -\mathcal{I}_{\rm c} \\ \mathcal{I}'_{\rm d} = -\mathcal{I}_{\rm d} \end{cases} , \tag{13}$$

where $K \equiv \mathcal{I}_{\rm b} + \mathcal{I}_{\rm c} + \mathcal{I}_{\rm d}$. If we consider $K = 0 \Rightarrow \mathcal{I}_{\rm a} = \mathcal{I}'_{\rm a}$, we note that the field cannot leak outside the torus because $K = \mathcal{I}_{\rm b} + \mathcal{I}_{\rm c} + \mathcal{I}_{\rm d} = 0 \Rightarrow \mathcal{I}_{\rm b} = \mathcal{I}_{\rm c} = \mathcal{I}_{\rm d} = 0$, since the field must go always in the same direction along these curves (the integrals have the same sign), hence $\mathbf{B}_{\rm b} = \mathbf{B}_{\rm c} = \mathbf{B}_{\rm d} = 0$ for our system ($\nabla \cdot \mathbf{B} = 0$). Therefore, the field along $C_{\rm a}$ and $C'_{\rm a}$ could be equal if both lines have the same length (case shown in Fig. S3(a), previously demonstrated). If they do not have the same length, the magnetic fields $\mathbf{B}_{\rm a}$ and $\mathbf{B}'_{\rm a}$ should be different but this cannot happen since the field does not leak the cavity. With this, we demonstrated that having asymmetric holes (Fig. S3(b)) implies $K \neq 0$, since initial premise is breached, so field lines will leak the cavity by one hole and reenter by the other resulting in different values for $\mathbf{B}_{\rm a}$ and $\mathbf{B}'_{\rm a}$.

From these results, we can generalize the conditions for which the magnetic field does

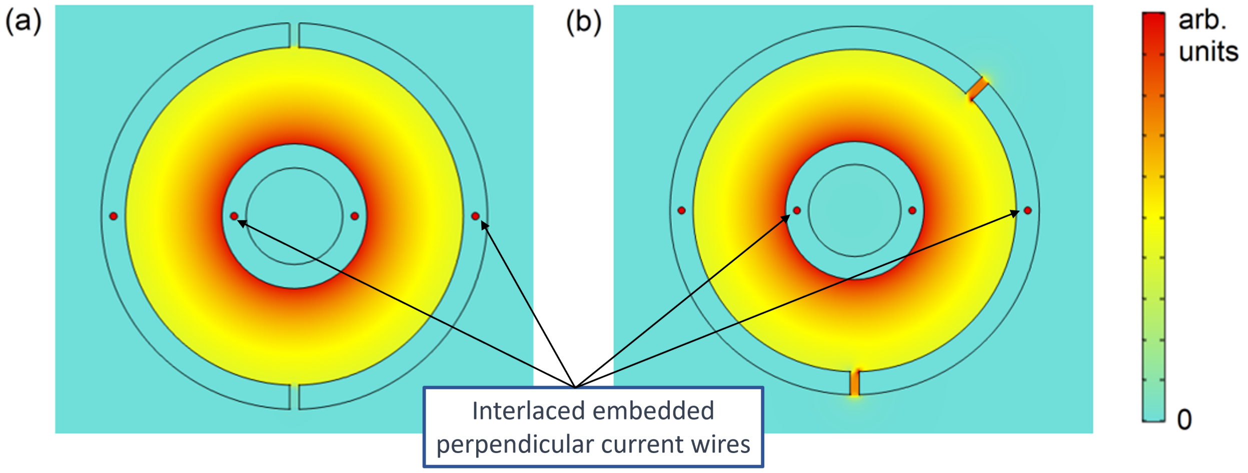

FIG. S4: Magnetic field strength output. (a), Symmetrical-drilled torus shown in Fig. S3(a). (b), Asymmetrical-drilled torus shown in Fig. S3(b). It is important to note that in (a) there is no field leakage while in (b) it leaks through the holes.

not leak out of the cavity, and consequently, this field does not lose its axisymmetry (as long as the holes are thin and long enough, as discussed above). In general, this happens for a system in which any Ampère's curves analogous to the ones in Fig. S3 have the same $\frac{I}{\Delta\theta}$ ratio (being I the intensity current threading the Ampère's curve and $\Delta\theta$ the toroidal angle defined by the sector limited by the two holes through which this curve passes). It is interesting to note that this solution, constant $\frac{I}{\Delta\theta}$, is independent of the number and position of the coils.

This leads to different possibilities in which the field does not leak, such as placing several holes in a transverse plane (any vertical plane defining one cross-sectional cut at a specific toroidal rotation, θ), among others.

In practice, the robustness of the preservation of the field in the cavity is maintained even though these conditions are not achieved exactly. For the ideal cases in which the field does not exit, small departures from these conditions of symmetry (e. g. small variations in the positions of the holes, fluctuations in the currents of the coils) do not cause a destabilization of the system, since to obtain clearly unstable situations a great rupture of the symmetry conditions would be necessary. In fact, very asymmetric systems such as the one in Fig. S4(b) only cause variations up to 1.3% in the $|\mathbf{B}_{\rm a}|$ and $|\mathbf{B}'_{\rm a}|$ values (where the cavity-hole diameters quotient, fraction between the diameter of the cavity and the diameter of the hole, is 10 and the longitude-diameter quotient of the holes, fraction between the length of the hole and the diameter of the hole, is 2.5). Moreover, even in very asymmetric cases,

field losses to the outside and the modification of the cavity field can be compensated by properly correcting and adjusting the currents in the different coils of the system.

Implementation in fusion magnets

As an example of the application of our ideas to fusion magnets, we consider the LHD configuration, where the inner toroidal cavity undergoes a rotational transform and the field has both toroidal and poloidal components. The previously described properties are kept if the above symmetry condition is maintained, as shown in Fig. S5. This would be true also for any regular cavity shape.

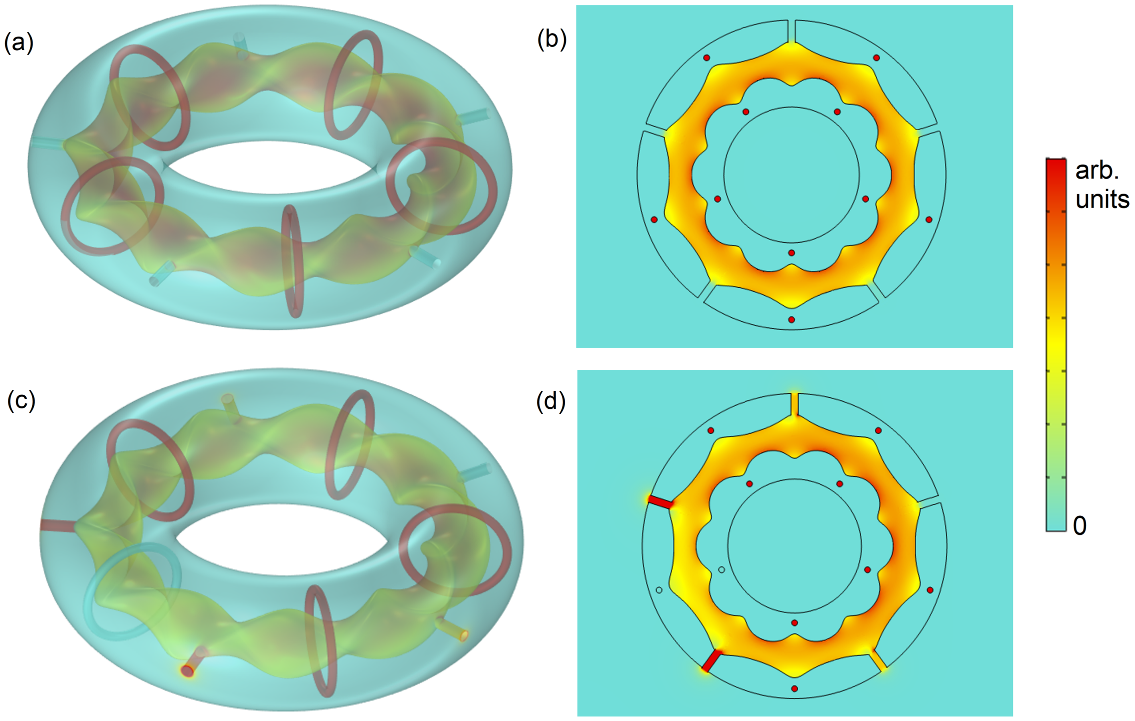

FIG. S5: Finite-element simulations of the 3D color map of the magnetic-field strength created by a superconducting toroid with a toroidal cavity, in which five holes to access the cavity from the exterior have been drilled. The configuration corresponds to the LHD experiment, as in Fig. 3. It can be observed that when holes are properly distributed as in (a), the magnetic flux leakage to the holes is practically zero, thus preserving the flux shape in the cavity. When the holes are not symmetric, as in (c), some flux is lost and the field in the cavity is modified. (b) and (d) are cross-sectional views of (a) and (c), respectively.

When taking into account the hole size it can be seen from Fig. S6 that the hole radius affects just locally to the magnetic field. Even for holes of the order of the cavity size, Fig. S6 (e) and (f), perturbations remain local and without affecting the cavity field globally.

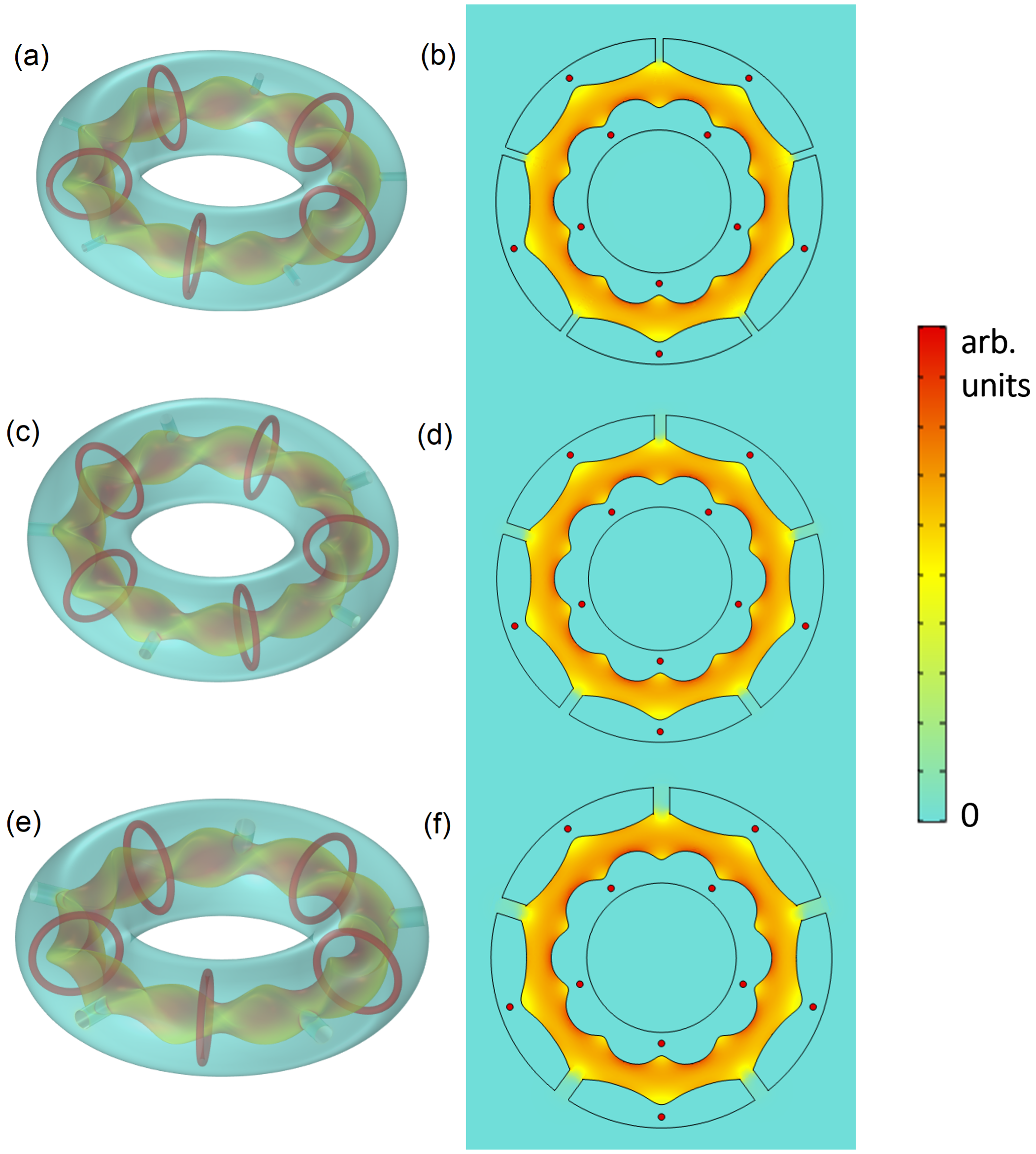

FIG. S6: Finite-element simulations of the 3D color map of the magnetic-field strength created by a superconducting toroid with a toroidal cavity, in which five holes had been drilled to access the cavity. Different hole radii have been drilled into (a), (c), and (e) with radii 0.05m, 0.075m, and 0.1m respectively. It is important to remark the elliptical cavity has a semi-minor axis length of 0.15m and a semi-major axis length of 0.3m. (b), (d), and (f) are horizontal cut planes of (a), (c), and (e) respectively. It can be observed that **B** decays very quickly along the hole and that local perturbations in the hole entrance are small.

The number of holes can be limited by maintaining constant the previously-mentioned quotient, $\frac{I}{\Delta\theta}$. Therefore, one possibility for increasing the number of holes is increasing the number of coils. In Fig. S7 we show an example, by doubling the number of coils and holes. Despite doubling the number of holes we see that the cavity field is maintained and there is

no field leakage.

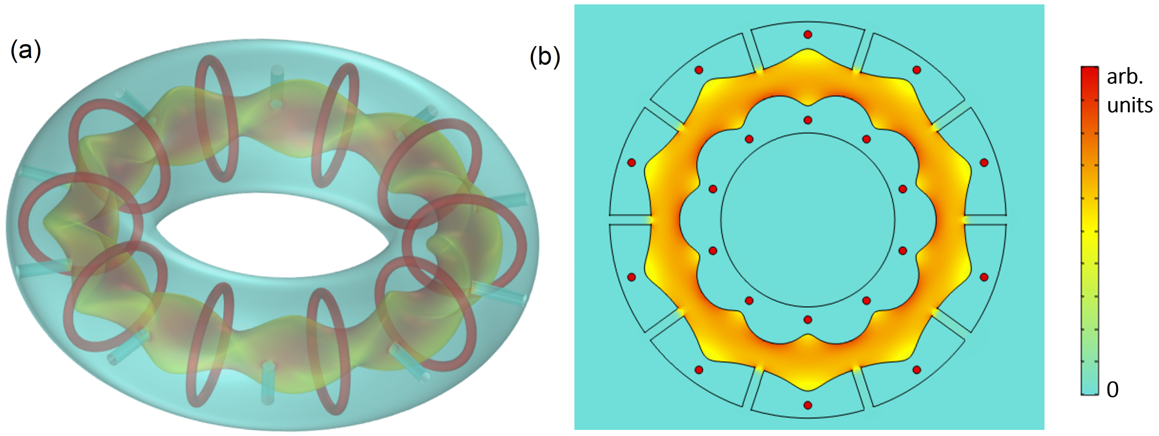

FIG. S7: Finite-element simulations of the 3D color map of the magnetic-field strength created by a superconducting toroid with a toroidal cavity, in which ten holes had been drilled to access the cavity. The number of current wires around the cavity is also ten. It can be observed that in the same way as for five holes and five currents no **B** threads outside the cavity. (b) is a horizontal cut plane of (a).

Another way of adding more holes to this kind of system is by placing a number of them in a transverse plane, as previously explained. This strategy allows us to access the plasma through more holes and without increasing the number of coils, preserving the magnetic flux surfaces of the cavity and avoiding field leakage [Fig. S8].

To sum up, in order to access the inner cavity there are several possible alternatives, either by playing with the size of the holes or by increasing the number of holes. In practice, a smart combination of all strategies would be the best way to access the plasma while maintaining flux surfaces and minimizing field leakage.

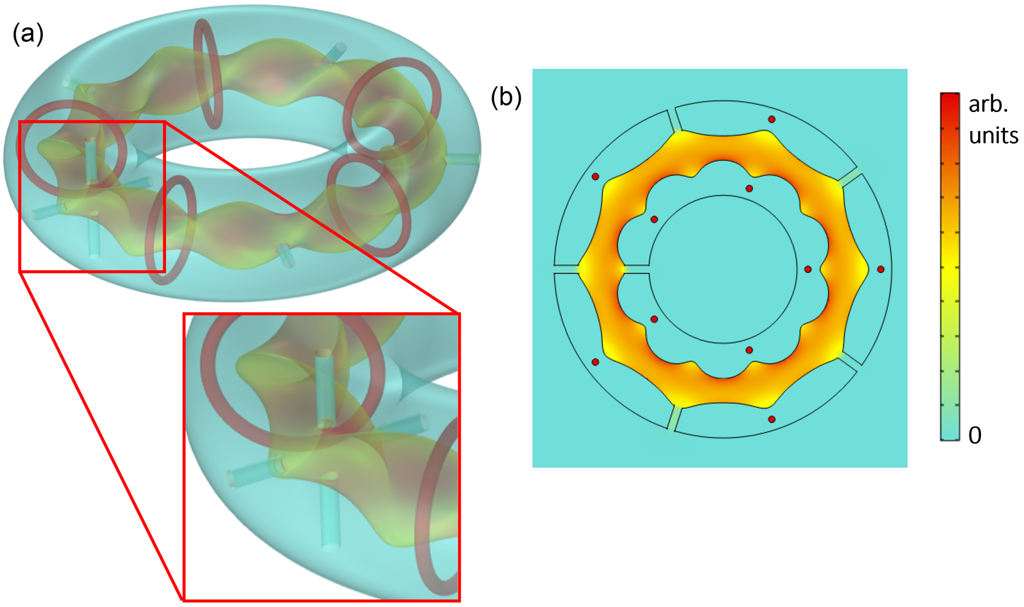

FIG. S8: Finite-element simulations of the 3D color map of the magnetic-field strength created by a superconducting toroid with a toroidal cavity. A total of eight holes had been drilled, one between each current loop except for one place where four holes had been drilled in the same transverse plane between two current loops. A zoom of the place where the four holes in the same transverse plane is provided. It can be observed that **B** does not exit outside the cavity through any hole. (b) is a horizontal cut plane of (a).
